# Supplementary material for: Hippocampal adult-born granule cells drive network activity in a mouse model of chronic temporal lobe epilepsy
Source: Nat Commun. 2020 Dec 1;11:6138. doi: 10.1038/s41467-020-19969-2 (PMC7708476; doi:10.1038/s41467-020-19969-2)
Supplement: Supplementary file 1 — Supplementary information [file 41467_2020_19969_MOESM1_ESM.pdf]

Supplemental Information

Figure S1

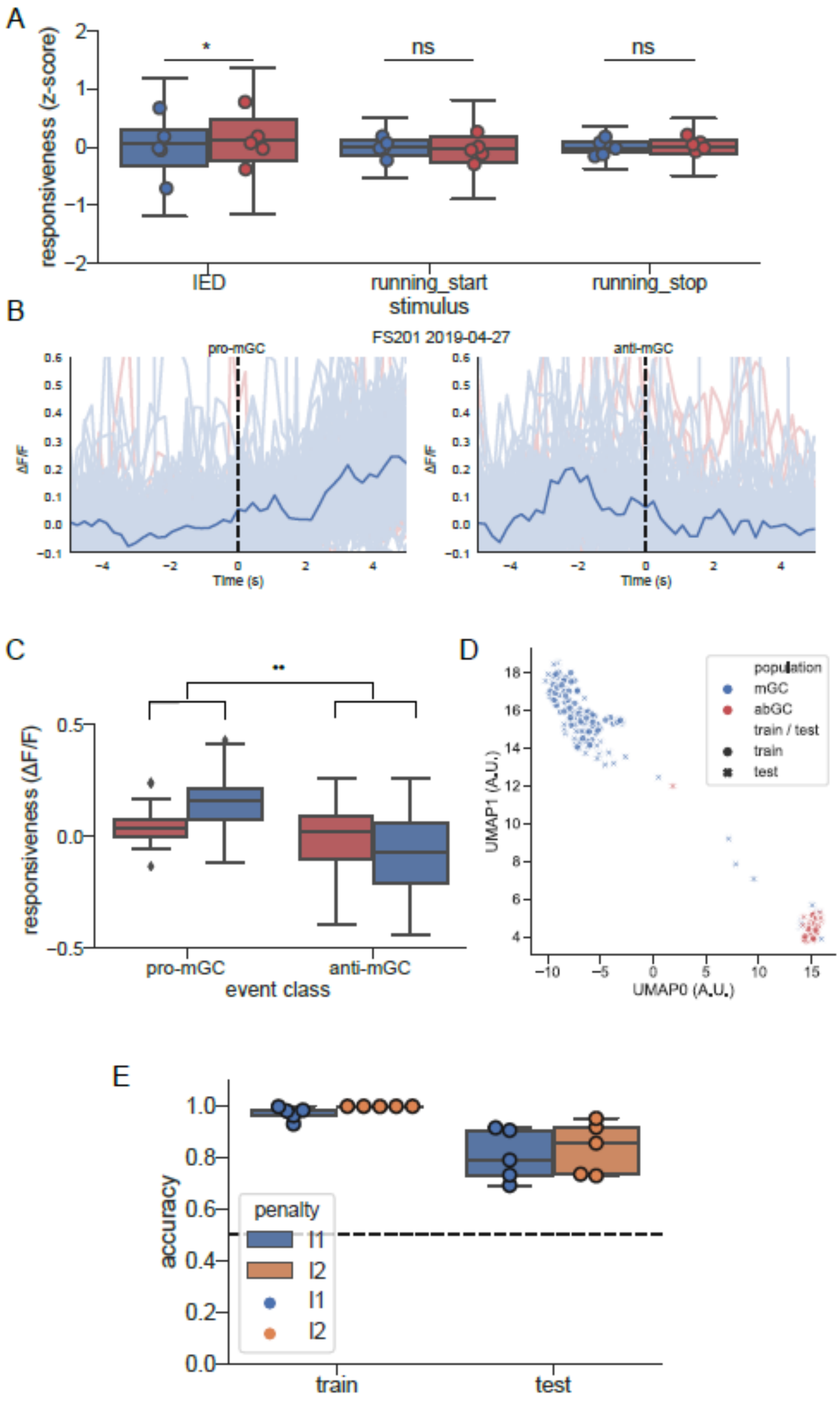

Figure S1. Responsiveness of abGC and mGC populations to locomotion and IEDs. A. Average *z*-scored $\Delta F/F$ response to locomotion onset, offset, and IED , where 'responsiveness' is defined as mean $\Delta F/F_{post}$ – mean $\Delta F/F_{pre}$ ('pre' and 'post' refer to 3 s windows pre/post event onset) (n = 5 mice, mGCs (n = 2162), abGC (*n* = 277). Center line, median; box limits, upper and lower quartiles; whiskers, 1.5x interquartile range. Each dot represents the average population response of one mouse. We find no significant difference in run-start (p = 0.89) or run-stop (p = 0.34) responsiveness between the abGC and mGC populations, but we do find that the abGC population is more responsive to IEDs compared to the mGC population (p = 0.04, Wilcoxon signed-rank test). **B.** Example PSTHs of single-cell responses to "most informative" IEDs from 10 min recording from one mouse. Single-cell responses to pro-mGC (left) and pro-mGC (right) events exhibit high levels of heterogeneity despite population trends. **C.** Population responses to pro-mGC and anti-mGC events identified in *Figure 3*. Center line, median; box limits, upper and lower quartiles; whiskers, 1.5x interquartile range, points, outliers. A two-way analysis of variance yielded a main effect for IED class (pro-mGC vs anti-mGC), $F(3,54) = 14.11$, $p=6\text{x}10^{-7}$, such that pro-mGC IEDs positively modulated mGCs compared to anti-mGC IEDs. The main effect of population was non-significant, $F(3,54)=0.13$, $p=0.71$, which suggests that our observations cannot be explained by intrinsic differences in responsiveness between the two populations independent of the type of IED. However, the interaction effect was significant, $F(3,54) = 10.29$, $p=0.002$, indicating a crossing over effect, i.e., that pro-mGC IEDs significantly positively modulated mGCs over abGCs. **D.** UMAP dimensionality reduction of mGC and abGC IED-response profiles from one session. A scalar responsiveness is calculated for each cell to each IED, and these vectors are nonlinearly embedded by UMAP into a 2D space for visualization. A subset of cells (crosses) was held out to test for consistency of the embedding. **E.** Cross-validated accuracy of logistic regression (Figure 3C) across animals. Center line, median; box limits, upper and lower quartiles; whiskers, 1.5x interquartile range; points, individual animals.

Figure S2

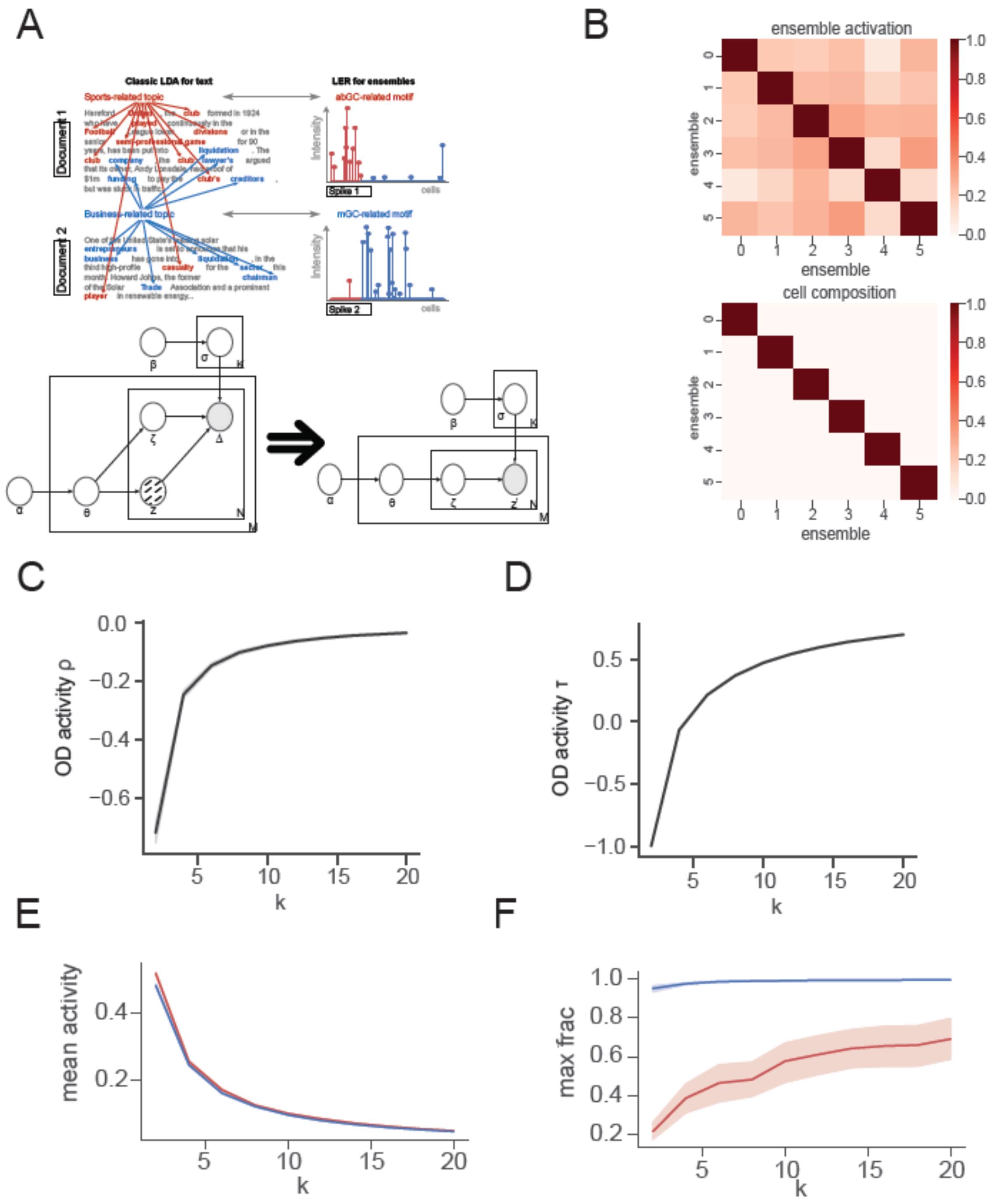

Figure S2. Ensemble statistics are conserved across animals. Quantification of the extent to which the ensemble structure learned for one mouse generalizes across animals. LER fit to data from n = 5 epileptic mice over a range of ensemble numbers *K*, with 10 realizations of LER fitted per K per mouse. **A.** Top: Analogy between Latent Dirichlet Allocation (LDA) for topics and LER for ensembles. LDA finds topics interpreted as “sports-related” or “business-related.” LER finds

ensembles of co-occurring neuronal activations that can be interpreted as “abGC-related” or “mGC-related”. Bottom: Base LER graphical model (left) and reduction to LDA (right) via point estimate of z (hatched). The principal difference between the models is that “words” (~cells) included in a “document” (~IED) are assumed to be certain by LDA but are uncertain and inferred by bootstrapping in our model. **B.** Characterization of learned ensembles and activity patterns, from one animal. Top: Ensemble activity patterns are weakly correlated (Kendall’s τ). Our model is able to accommodate the joint activation of multiple ensembles in a single event; this observation suggests that weak inter-ensemble correlation is present in our data. Bottom: Ensembles are close to orthogonal (Pearson’s correlation on cell weights), suggesting the epileptic network organizes into *disjoint* pathologic ensembles. **C.** Average off-diagonal correlations by cell pooled across mice (Pearson’s ρ). Except when K is small (<4), off-diagonal entries in the cell correlation matrix near 0. **D.** Average off-diagonal correlations by activity pooled across mice (Kendall’s τ). Except when K is small, off-diagonal entries in the cell correlation matrix are weakly positive. **E.** Mean normalized activity of abGC- vs mGC-dominated ensembles, plotted as a function of K. **F.** The “purity” of the “purest” abGC ensemble to the “purest” mGC ensemble, as a function of K. This “maximum purity” (expressed as a fraction from 0 to 1) is increasing with K for both populations, but nearly pure (>90%) mGC ensembles can be identified regardless of K, whereas the purest abGC ensemble only contains 80% abGCs at K=20, suggesting that certain mGCs are highly coupled to abGCs (data presented as mean value +/- 95% CI).

Figure S3

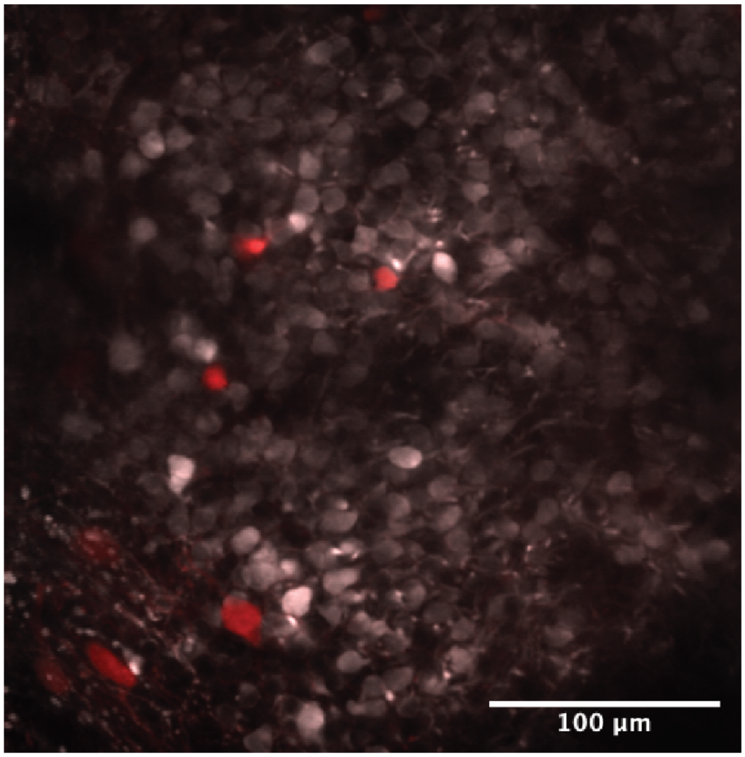

Figure S3. Minimal number of interneurons imaged in GCL. Example *in vivo* two-photon field of view from GCL in a Vgat-Cre x Ai9-tdTomato mouse. Although a rAAV-driven expression of GCaMP labels both excitatory and inhibitory populations in the DG, excitatory neurons form the overwhelming majority of the imaged population in this study (GCaMP = white; tdTomato = red). To quantify the potential impact of GABAergic neurons labeled among the DG granule cells in the KA model, we imaged GCL in a Vgat-Cre x Ai9-tdTomato mouse line, finding that GABAergic neurons constitute less than 1% of the neurons in a typical FOV.

Figure S4

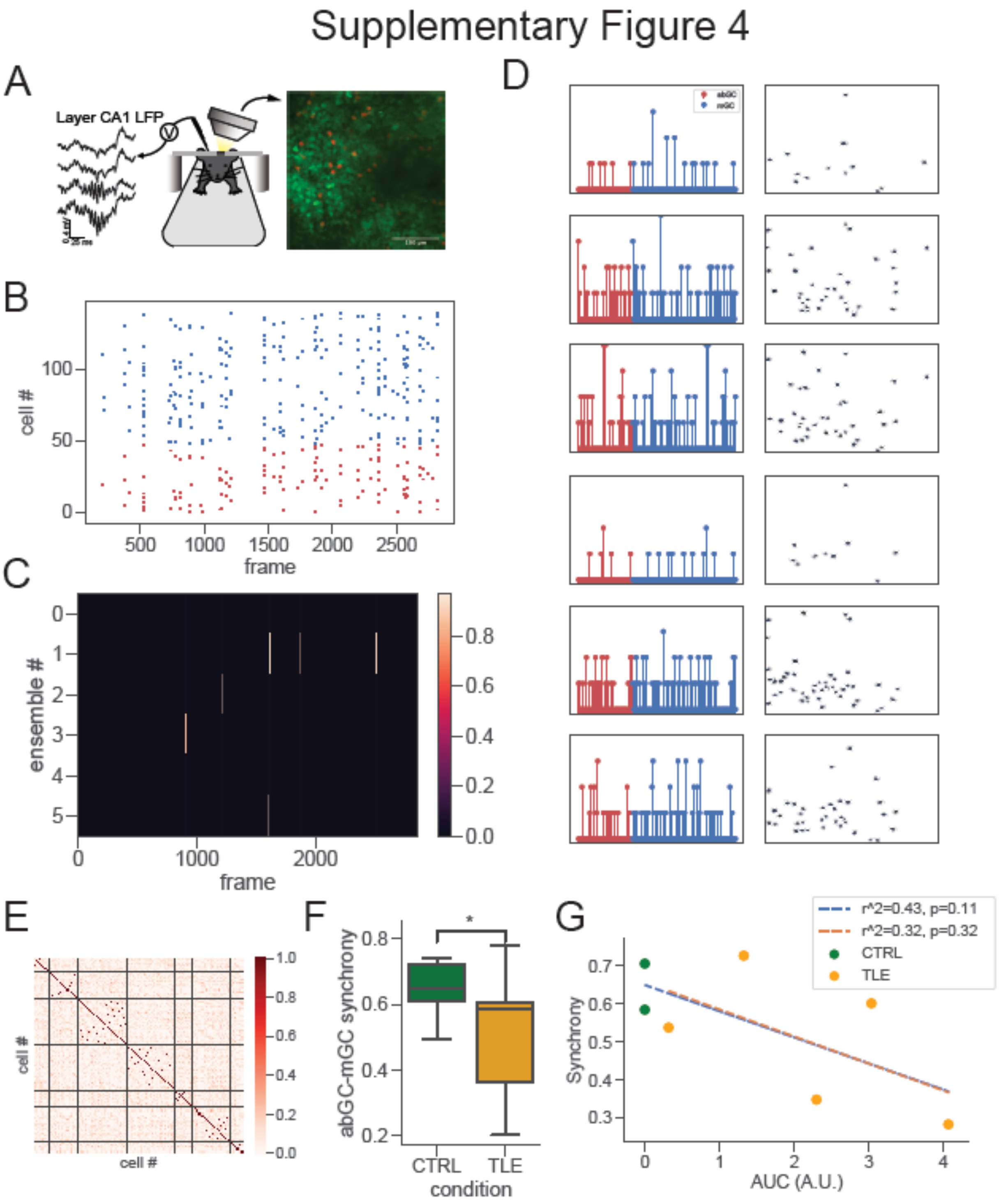

Figure S4. Ensemble recruitment by sharp wave ripples in control animals. A. Schematic of simultaneous 2p imaging and SPW-R recording (n=2 mice). **B.** Binarized activation matrix with respect to SPW-Rs, using the same binarization procedure as in Figure 4B. **C.** Ensemble activity

matrix shows sparse activation of ensembles. **D.** Learned ensembles in the network as in Figure 4D. The non-epileptic DG does not segregate into abGC and mGC ensembles; instead, all inferred ensembles are mixed, suggesting that physiological microcircuits use the two populations to perform ripple-associated computations in a synchronized manner, which is disrupted in IEDs in TLE. **E.** Cell-cell correlation matrix, sorted by ensemble ID. Cells tend to be correlated with other cells in the same ensemble, while having low correlation with cells in different ensembles. **F.** abGC event responses are significantly desynchronized from mGCs event responses in TLE. Synchrony is defined as the Kendall tau correlation between event-triggered activations in each population (two-sided Mann-Whitney *U*-test on N_{TLE}=17 IED recording sessions from 5 mice, N_{CTRL}=8 SPW-R recording sessions from 2 mice, p = 0.017). Error bars: 95% confidence interval for mean, calculated from 1000x bootstrap sampling on recording sessions with replacement. **G.** Seizure AUC taken as the integral of an indicator variable representing seizure occurrence over the most proximal 24 hour period to the imaging session (10 days prior to imaging), that corresponds to the seizure frequency weighted by seizure duration, 1 point = 1 animal. Regression lines fit to either IED synchrony only (orange dashed line) or event (IED or SPW-R) synchrony (blue dashed line, including non-epileptic mice).

Table S1. Per subject Suite2p extracted ROIs

	mGC	abGC	Total	% abGC
FS201	142	39	181	21.5
FS202	218	48	266	18.0
FS203	428	39	467	8.4
FS204	763	41	804	5.1
FS205	613	111	724	15.3
**Total**	2164	278	2442	
**Mean**	432.8	55.6	429.5	13.3
**SD**	260.9	31.2	273.6	6.8
**N**	5	5	5	5
**SEM**	116. 7	13.9	122.4	3.1
