# Supplementary material for: Hippocampal adult-born granule cells drive network activity in a mouse model of chronic temporal lobe epilepsy
Source: Nat Commun. 2020 Dec 1;11:6138. doi: 10.1038/s41467-020-19969-2 (PMC7708476; doi:10.1038/s41467-020-19969-2)
Supplement: Supplementary file 3 — Reporting Summary [file 41467_2020_19969_MOESM3_ESM.pdf]

nature research

Corresponding author(s): Attila Losonczy

Last updated by author(s): Oct 11, 2020

Reporting Summary

Nature Research wishes to improve the reproducibility of the work that we publish. This form provides structure for consistency and transparency in reporting. For further information on Nature Research policies, see our Editorial Policies and the Editorial Policy Checklist.

Statistics

For all statistical analyses, confirm that the following items are present in the figure legend, table legend, main text, or Methods section.

n/a	Confirmed	
[ ]	[x]	The exact sample size (n) for each experimental group/condition, given as a discrete number and unit of measurement
[ ]	[x]	A statement on whether measurements were taken from distinct samples or whether the same sample was measured repeatedly
[ ]	[x]	The statistical test(s) used AND whether they are one- or two-sided<br>*Only common tests should be described solely by name; describe more complex techniques in the Methods section.*
[ ]	[x]	A description of all covariates tested
[x]	[ ]	A description of any assumptions or corrections, such as tests of normality and adjustment for multiple comparisons
[ ]	[x]	A full description of the statistical parameters including central tendency (e.g. means) or other basic estimates (e.g. regression coefficient) AND variation (e.g. standard deviation) or associated estimates of uncertainty (e.g. confidence intervals)
[ ]	[x]	For null hypothesis testing, the test statistic (e.g. F, t, r) with confidence intervals, effect sizes, degrees of freedom and P value noted<br>*Give P values as exact values whenever suitable.*
[ ]	[x]	For Bayesian analysis, information on the choice of priors and Markov chain Monte Carlo settings
[x]	[ ]	For hierarchical and complex designs, identification of the appropriate level for tests and full reporting of outcomes
[ ]	[x]	Estimates of effect sizes (e.g. Cohen's d, Pearson's r), indicating how they were calculated

Our web collection on statistics for biologists contains articles on many of the points above.

Software and code

Policy information about availability of computer code

Data collection	The software used to collect data in this study was: 1) Prairie View (Bruker) for two-photon imaging; 2) custom Matlab (verson R2011b) software for continuous video-EEG; 3) RHD Recording Controller (Intan) for LFP recording during imaging.
Data analysis	The data analysis for this study was mainly performed using custom Matlab R2019a and Python 2.7 code and the open source packages scikit-learn and SIMA, which are available at https://github.com/scikit-learn/scikit-learn and https://github.com/losonczylab/sima/. Custom code written for this study is available at https://github.com/losonczylab.

For manuscripts utilizing custom algorithms or software that are central to the research but not yet described in published literature, software must be made available to editors and reviewers. We strongly encourage code deposition in a community repository (e.g. GitHub). See the Nature Research guidelines for submitting code & software for further information.

Data

Policy information about availability of data

All manuscripts must include a data availability statement. This statement should provide the following information, where applicable:

- Accession codes, unique identifiers, or web links for publicly available datasets
- A list of figures that have associated raw data
- A description of any restrictions on data availability

The datasets generated and analyzed during the current study are available in the Dyad Digital Repository https://doi.org/10.5061/dryad.tx9569w1.

Field-specific reporting

Please select the one below that is the best fit for your research. If you are not sure, read the appropriate sections before making your selection.

- [x] Life sciences
- [ ] Behavioural & social sciences
- [ ] Ecological, evolutionary & environmental sciences

For a reference copy of the document with all sections, see nature.com/documents/nr-reporting-summary-flat.pdf

Life sciences study design

All studies must disclose on these points even when the disclosure is negative.

Sample size	Sample size was determined based on prior experience, previous studies including variability of epileptogenesis (Marchionni et al., 2018), electrode implant, viral injection, calcium indicator expression and imaging window quality (Danielson et al., 2016, Zaremba et al., 2017, Turi et al., 2019, Kaufman et al., 2020) and our discussion with other experts. Efforts were made to keep the number of animals used at a minimum to accomplish unequivocal testing of the hypotheses and achieve statistical significance.
Data exclusions	No data was excluded from the analyses.
Replication	Using the set of experimental parameters detailed in our methods to examine neural ensemble structure in temporal lobe epilepsy, we find distinct organization within mature and adult-born dentate gyrus granule cell populations. To verify reproducibility, we performed the same analyses across all of our experimental subjects, and we found that the organizational properties of these cell populations in TLE are consistent and conserved across all individual animals within our study.
Randomization	Randomization of experimental groups was not required as there is only one group.
Blinding	Blinding to group allocation was not required as there was only one group. For training of the local field potential interictal epileptiform discharge classifier, the researchers were blind to the corresponding functional calcium imaging data.

Reporting for specific materials, systems and methods

We require information from authors about some types of materials, experimental systems and methods used in many studies. Here, indicate whether each material, system or method listed is relevant to your study. If you are not sure if a list item applies to your research, read the appropriate section before selecting a response.

Materials & experimental systems

n/a	Involved in the study
x	Antibodies
x	Eukaryotic cell lines
x	Palaeontology and archaeology
	x Animals and other organisms
x	Human research participants
x	Clinical data
x	Dual use research of concern

Methods

n/a	Involved in the study
x	ChIP-seq
x	Flow cytometry
x	MRI-based neuroimaging

Animals and other organisms

Policy information about studies involving animals; ARRIVE guidelines recommended for reporting animal research

Laboratory animals	Male transgenic mice were obtained from The Jackson Laboratory to establish a local breeding colony on a C57BL/6J background: Nestin-CreERT2 (JAX:016261) and ROSA26-CAG-stopflox-tdTomato Ai9 (JAX:007909). The Nestin-Cre line was crossed with the Ai9 reporter line to express tdTomato in adult-born granule cell populations. Mature male and female mice (>8 weeks of age) were used for all experiments. Mice were housed in the vivarium on a 12h light/dark cycle (lights on at 07:00), constant temperature (21-24C) and humidity (30-50%), were housed 3-5 mice per cage, and had access food and water ad libitum.
Wild animals	No wild animals were used.
Field-collected samples	No field-collected samples were used.
Ethics oversight	All experiments were conducted in accordance with the US National Institutes of Health guidelines and with the approval of the Columbia University Animal Care and Use committee

Note that full information on the approval of the study protocol must also be provided in the manuscript.
